# Human and Mouse Alpha-Synuclein Fibrillation: Impact on h-FTAA Binding and Advancing Strain-Specific Biomarkers in PD Animal Models

**DOI:** 10.3390/ijms27093807

**Published:** 2026-04-24

**Authors:** Priyanka Swaminathan, Vasileios Theologidis, Hjalte Gram, Debdeep Chatterjee, Per Hammarström, Nathalie Van Den Berge, Mikael Lindgren

**Affiliations:** 1 Department of Physics, Faculty of Natural Sciences, Norwegian University of Science and Technology (NTNU), Gløshaugen, Realfagbygget, NO-7491 Trondheim, Norway; priyanka.swaminathan@ntnu.no; 2 Department of Clinical Medicine—Core Center for Molecular Morphology, Section for Stereology and Microscopy, Aarhus University, 8000 Aarhus, Denmarknathalie.vandenberge@clin.au.dk (N.V.D.B.); 3 Danish Research Institute of Translational Neuroscience (DANDRITE), Department of Biomedicine, Aarhus University, 8000 Aarhus, Denmark; hjalteg@clin.au.dk; 4 PACE—Lundbeck Foundation Parkinson’s Disease Research Center, Aarhus University Hospital, 8200 Aarhus, Denmark; 5 Department of Physics, Chemistry and Biology, Linköping University, SE-581 83 Linköping, Sweden; debdeep.chatterjee@liu.se (D.C.); per.hammarstrom@liu.se (P.H.); 6 Science for Life Laboratory, Linköping University, SE-581 83 Linköping, Sweden

**Keywords:** αsyn aggregation kinetics, αsyn rodent model, h-FTAA binding, FLIM

## Abstract

Disease-specific alpha-synuclein (αsyn) strains have been linked to different synucleinopathies. Current αsyn biomarkers are limited to binary detection of pathogenic αsyn in peripheral tissue biopsies or fluids, limiting differential diagnosis. Hence, there is an urgent need for methods that allow strain-specific detection and characterization of αsyn strain architecture. Notably, luminescent conjugated oligothiophenes (LCOs) have been successfully used to detect distinct protein strain conformers in prion diseases and Alzheimer’s disease, highlighting their utility in differentiating disease-specific amyloid structures. Species-dependent differences in αsyn structure are increasingly recognized as one of the critical aspects that shape how fibrils form, propagate and interact with molecular LCO probes. Here, we evaluate the potential of the LCO h-FTAA to differentiate species-specific αsyn strains and conduct a translational investigation using peripheral cardiac tissue of a gut-first synucleinopathy rodent model. Our in vitro data demonstrate strain-specific probe–fibril interactions, reflecting a differential strain architecture and cellular micro-environment. While h-FTAA binds with comparable efficiency to mouse (mo-) and human (hu-) pre-formed fibrils (PFFs), h-FTAA exhibits markedly lower quantum yield when bound to moPFFs versus huPFFs. Spectral imaging revealed h-FTAA-moPFF binding produces blue-shifted maxima (505–550 nm), contrasting with the red-shifted maxima (545–580 nm) of huPFFs. Fluorescence lifetime imaging microscopy confirmed h-FTAA’s intrinsic sensitivity to species-dependent variations through distinct temporal fluorescence signatures (moPFFs: ~0.60–1.5 ns vs. huPFFs: ~0.65–1.0 ns). Our translational investigation showed h-FTAA binding to peripheral cardiac pathology exhibits comparable red-shifted emission, but distinct fluorescence lifetimes of h-FTAA-bound aggregates in moPFF-injected (~1.0–1.4 ns) versus huPFF-injected (~0.69–0.8 ns) rats. Interestingly, we observed distinct blue-shifted emission profiles in a few selected regions of the heart of moPFF-injected rodents, further characterized by extra-long fluorescence decay shifts (~1.5–1.9 ns), reflecting differences in both aggregate conformation and maturity in moPFF-induced compared with huPFF-induced rats. Taken together, our findings underscore the potential of LCO ligands, like h-FTAA, to enable more precise disease staging and diagnosis through peripheral biopsies, complementing existing αsyn biomarker methods.

## 1. Introduction

The buildup of αsyn into fibrils, eventually forming pathogenic inclusions or filaments, is a delineated feature of several synucleinopathies, including Parkinson’s disease (PD), dementia with Lewy bodies (DLB), pure autonomic failure (PAF) and multiple system atrophy (MSA). Most importantly, αsyn protein fibrillation is a complicated multistep process emerging from the interplay of several molecular processes, including conformational variabilities in monomers, protein nucleation, and fibril elongation, followed by secondary amplification mechanisms that collectively shape the rate and kinetics of αsyn fibrillation [1,2]. The kinetics of these molecular processes are influenced by protein sequences, solution conditions such as pH, ionic strength, and buffer compositions [2]. Notably, the protein sequence strongly determines the fibrillation dynamics, whereby even a single change in the protein sequence alters/accelerates the process of αsyn aggregation. For instance, one of the point mutations, A53T of human αsyn sequence (huA53T), linked to familial early onset of PD is known to accelerate oligomerization and fibrillation in vitro as well as in animal models [3,4]. In another instance, αsyn generated from mouse species (mo-αsyn) and human αsyn (hu-αsyn) exhibits a high sequence identity of ~95%; however, the mo-αsyn differs by only seven amino acid residues. This includes residue 53, where the mouse protein carries the A53T substitution within its sequence, exhibiting faster fibrillation rates than the human αsyn [5,6,7].

Alpha-synuclein has been detected in the peripheral nervous system of prodromal patients up to 20 years before clinical diagnosis. In prodromal patients with autonomic dysfunction, αsyn is hypothesized to originate in the gut (PD/DLB) or in the urinary tract (MSA), driving the autonomic phenotype during the pre-motor phase [8,9]. In PD patients without autonomic dysfunction in the pre-motor disease stage, αsyn is hypothesized to originate in the amygdala or olfactory bulb [10]. However, direct evidence between the αsyn onset site and disease phenotype remains to be elucidated. The αsyn fibril model is commonly used to investigate αsyn onset and subsequent propagation and neural system dysfunction in animals. In both brain-first and gut-first seeding studies, hu-αsyn fibrils display low seed competence and fail to induce robust neurodegeneration in wild-type rodent models and in vitro ([10,11,12,13,14]). Likewise, mo-αsyn cross-seeding to hu-αsyn also confers low pathogenicity compared with αsyn seeding between homologous species [12]. Protein sequence differences attributed to asymmetric seeding may affect fibrillation kinetics in a way that could produce distinct fibril conformers [15].

Traditional methods such as thioflavin T (ThT) fluorescence provide robust measures of fibrillation kinetics by producing a characteristic increase in fluorescence intensity upon binding to cross-β-sheet structures [16,17]. This is the standard procedure for detecting mature amyloid fibrils, such as, for example, aggregated αsyn in PD and Aβ variants of Alzheimer’s disease (AD) [17,18]. But they lack sensitivity to detect subtle conformational differences and are limited in biological contexts due to poor cell penetration. Because differences in sequence compatibility may drive the formation of structurally distinct fibril conformers, tools capable of distinguishing these morphologies are essential. To this end, luminescent conjugated oligothiophenes (LCOs) have been characterized that provide morphology-sensitive spectral readout that reflects fibril architecture [19]. The LCOs have been widely applied to monitor fibrillation kinetics of some of the amyloid protein species, including αsyn, and are reported to capture early protein fibrillar species with greater sensitivity than ThT [20,21,22]. The heptameric LCO heptameric-formyl thiophene acetic acid (h-FTAA) has been used to distinguish αsyn aggregate morphologies in PD and MSA brain tissue sections and has similarly been used in in vitro cell models to resolve intracellular αsyn aggregates based on its conformation-sensitive optical readout [23,24]. It is also known for a notorious sensitivity to early aggregates of both Aβ and αsyn [19,21,22].

Isolated REM sleep behavior disorder (iRBD) is a key prodromal marker of synucleinopathies as patients convert to PD, DLB, MSA or PAF within 5–15 years of iRBD diagnosis [25]. Currently, MIBG scintigraphy of the heart is the only available method to differentiate PD/DLB from MSA in the iRBD-positive prodromal phase. However, this technique alone is insufficient for definitive diagnosis and is limited by accessibility [26]. The detection of αsyn in peripheral tissues, particularly through skin biopsies, has emerged as a highly promising diagnostic tool for synucleinopathies during the early iRBD stage, where peripheral pathology is more pronounced and detectable than in the manifest disease phase [27]. Importantly, it has been hypothesized that synucleinopathies are driven by distinct αsyn strains contributing to their clinical heterogeneity. Therefore, sensitive detection and characterization of peripheral αsyn aggregates using probes such as h-FTAA hold significant potential for subtype-specific diagnosis via minimally invasive peripheral biopsies [28]. Such an approach would enable earlier and subtype-specific diagnosis, improved disease stratification, and enhanced monitoring of progression and therapeutic response.

In this study, we characterize the fibrillation kinetics of recombinant mo- and hu-αsyn using ThT fluorescence, and we investigate the potential of h-FTAA spectral profiling to sensitively detect and distinguish mo- and hu-αsyn conformers in vitro. We further examine the translational potential of the h-FTAA probe as a peripheral biomarker by characterizing peripheral h-FTAA-labeled αsyn aggregates in gut-first PD rats following peripheral administration of mo- or hu-αsyn pre-formed fibrils (PFFs).

## 2. Results and Discussion

### 2.1. Kinetics of Aggregating huWT- and moWT-αsyn Monitored with ThT

**Figure 1:**
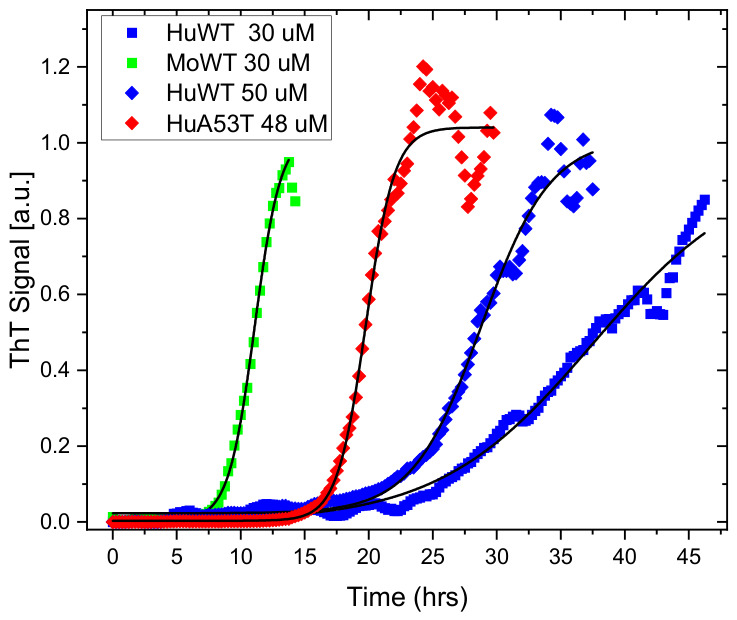
Kinetics of synuclein aggregation: huWT- (blue squares) and moWT-αsyn (green) at 30 μM, together with data of 50 μM of huWT (blue diamonds) and 48 μM of huA53T (red diamonds) redrawn from our earlier study for comparison [21]. ThT (10 μM) was used as a fluorescent amyloid ligand. The spectral emission was measured at time points every 15th minute, and the integrated signal is presented, normalized to the fitted plateau value in the plots. The samples were excited at λ_ex_ = 450 nm, and emissions were collected in the range λ_em_ = 470–620 nm. The black solid lines are fits described in the text and Section 3.3.

A fluorescence plate reader was utilized to investigate the fibrillation kinetics of αsyn in vitro using the amyloid-specific fluorescent ligand ThT. Human (huWT) and mouse (moWT) wild-type αsyn, along with PFFs for seeding, are described in Section 3.1. In the ThT assay, fibrils were produced by incubating monomeric protein (30 μM) under agitation at 37 °C for 40 h, as described in detail in Section 3.2. The time lapse curves presented in Figure 1 are typical for ThT fibrillation kinetics. Notably, the aggregation of mature fibrils was fastest for the moWT, as expected (green squares; $t_{1/2}=11\;h$), compared with the human variant (blue squares; $t_{1/2}=37.5\;h$) [17,18].

Added to the plot are also the data from our previously reported experiments, using huWT (blue diamonds; $t_{1/2}=29\;h$) together with human A53T (red diamonds; ($t_{1/2}=20\;h$) mutations, both based on 50 μM of native αsyn [21]. Both displayed kinetics intermediate between moWT and huWT aggregated at 30 μM, with A53T having an earlier onset and higher apparent rate compared with huWT, as reported by Sokratian et al. [15]. It should be noted that these time lapses are sensitive to temperature, protein concentration, salt [29], and the amount of ThT, although the latter effect is observed only at significantly higher ThT concentrations than those used in this study [30]. The solid black lines in Figure 1 represent fits to the aggregation time traces with the widely used two-parameter sigmoidal function, where the essential parameters $k_{app}$ (apparent growth rate) and $t_{1/2}$ (half-time) are found from the curve shape, and $t_{lag}$ (lag-time) is deduced from those two parameters, as summarized in Table 1 (see Section 3.3 for the assessment details).

### 2.2. Seeding Kinetics of huWT- and moWT-αsyn PFFs Monitored with ThT

**Figure 2:**
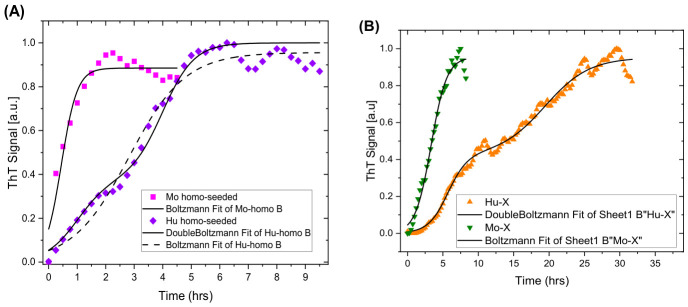
Real-time fibrillation kinetics monitored by ThT fluorescence (**A**) Homo-seeding: huWT-αsyn seeded with huWT PFF (blue diamonds) and moWT-αsyn seeded with moWT PFF (purple squares). (**B**) Cross-seeding: huWT-αsyn seeded with moWT PFF (orange triangles) and moWT-αsyn seeded with huWT PFF (green triangles). In all cases, 10% (wt) of PFFs were added at the start. ThT (10 μM) was used as a fluorescent amyloid ligand. The spectral emission was measured at time points every 15th minute, and the integrated signal is presented. The samples were excited at λ_ex_ = 450 nm, and emissions were collected in the range λ_em_ = 470–620 nm. Black solid lines are fits described in the text and Section 3.3.

The kinetics of αsyn aggregation is well known to be enhanced upon seeding the native protein with PFFs, both by seeding with the same PFF mutation (homo-seeding) as well as when seeding with mutated PFFs or other species of protein aggregates (cross-seeding) [12,31]. The results of such seeding fibrillation are presented in Figure 2. The homo-seeding processes progress much faster (Figure 2A) than any of the cross-seeding experiments (Figure 2B). In fact, the fibrillation is so enhanced that the lag phase cannot be resolved/observed for native moWT seeded with moWT fibrils (magenta squares). Thus, aggregation progresses distinctly without the need for the creation of nucleation sites.

In the cross-seeding experiment, the native huWT seeded with moWT fibrils is much slower (Figure 2B, orange triangles) in comparison with the non-seeded fibrillation of huWT (Figure 1, blue squares). Similarly, moWT seeded with native huWT does not enhance the aggregation kinetics (Figure 2B, green triangles). This is in agreement with earlier findings on the restricted bidirectionality between mouse and human PFF seeding [12].

Interestingly, both cases of seeded huWT fibrils resolve a biphase process. This is especially evident in the case of cross-seeding with moWT onto native huWT (Figure 2B, orange triangles). Here, the aggregation curves were fitted to the sigmoidal model, allowing for a biphasic process in the cases of human PFF as the seeding target (Table 2; see Section 3.3 for details of the assessment). Due to the absent, or very short, lag-phase, only the apparent aggregation rate and the half-time were included. For the biphase model, many parameters are needed that bring uncertainty into the parameters. Here, the apparent half-times were decided by visual inspection of the relevant plots and held constant in the fitting of the parameters $k_{app}$ and $t_{1/2}$.

The observed kinetic differences between moWT and huWT αsyn reflect fundamentally distinct fibrillation processes. HuWT-αsyn follows biphasic kinetics with separate nucleation and growth phases, potentially creating a window where toxic oligomeric species accumulate. In contrast, moWT-αsyn exhibits monophasic kinetics, suggesting a faster conversion from potentially harmful species into stable, less toxic mature fibrils. This efficient transition minimizes cellular exposure to damaging intermediates, creating a protective mechanism that would explain the inability of wild-type mice to develop spontaneous synucleinopathy, despite containing sequence elements that would accelerate aggregation in humans. However, the faster kinetics of mo-αsyn would drive faster fibril formation and propagation when introduced as external PFFs, yielding a more rapid peak aggregation compared with huPFFs in mouse seeding studies.

### 2.3. EM Imaging the Morphology of huWT and moWT PFFs

**Figure 3:**
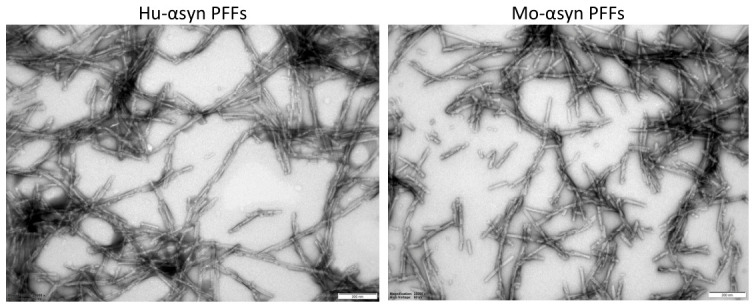
TEM images of huWT PFFs and moWT PFFs at 25,000× magnification; scale bar is 200 nm. TEM images at a lower magnification, 5000×, are presented in Figure S1.

Transmission electron microscopy images of the mouse and human PFFs were recorded (Section 3.4) to verify fibril structure and to compare the two different fibril types. Both PFFs showed canonical amyloid fibrillar morphologies with obvious similarities (Figure 3). PFFs were sonicated after fibril formation to generate higher amounts of free fibril ends by fragmentation.

There was a tendency for more short fibril fragments from the moPFF, indicating a higher fragmentation sensitivity of the moPFF (Figure 3 and Figure S1). The observed difference in fragment size was corroborated by dynamic light scattering (DLS; see Section 3.5). The moPFF fragments had an average hydrodynamic radius of 25.7 nm, while huPFF had an average radius of 55.2 nm (Figure S2). This difference in mo- and huPFF fragment sizes may impact the seeding activity of the PFF, as an increased number of fibril ends provides more nucleation sites and may template a faster seeding.

### 2.4. Binding Curves of h-FTAA with PFFs and Native αsyn

**Figure 4:**
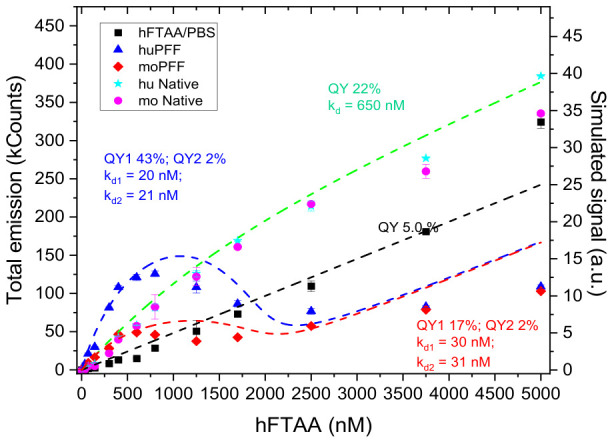
Binding curves of h-FTAA binding to moPFFs (red) and huPFF (blue)… λ_ex_ = 470 nm and emissions collected in the range λ_em_ = 490–750 nm. Black dashed lines are simulations described in the text and Section 3.6. Key-parameters are included as insets, color-coded with respective simulation (dashed curves).

To investigate the spectral response and enhanced fluorescence for h-FTAA binding with αsyn PFFs, so-called binding assays were prepared (for details, see Section 3.6). A fixed concentration of protein (1 μM) was exposed to various concentrations of h-FTAA ranging between 0 and 5 μM. We prepared samples with initially smaller concentration steps and a more extended range of the ligand dye concentration compared with our previous study [23] to resolve more details of the binding sites. Also, the apparent binding to the native (non-fibrillated) huWT and moWT variants was included. Compared with our previous experiment, the excitation wavelength was set to be somewhat longer, 470 nm, and after appropriate background corrections, the emission spectra were integrated for the whole concentration range. The resulting binding curves are plotted in Figure 4.

The blue triangles and the red diamonds represent the huWT and moWT PFFs, respectively, displaying markedly different fluorescence emission profiles despite similar binding affinities. Upon initial binding, fluorescence emission increases for both variants, with huWT showing a significantly sharper rise compared with moWT. This is followed by a notable decrease in fluorescence at ligand concentrations higher than approximately 1 µM. This behavior is typical for closely located, nearly identical binding sites, similar to what has been observed with small hydrophobic molecules binding to tetrameric transthyretin (TTR). In TTR, binding of a second fluorescent ligand (X34, a Congo Red derivative) quenches the emission of the entire system, as demonstrated by Sundnes et al. [32]. Similar phenomena could also occur for larger fibrillar structures, especially as both the human and mouse PFFs are known to form rigid structures with two-fold or pseudo-two-fold symmetry axes ([15,33,34,35]), implying pairwise identical binding sites.

The dashed curves in Figure 4 are simulations where a two-site model was adopted for the huWT and moWT binding. Each curve contains the contribution from each binding site in terms of the dissociation constant of each, to yield the total fluorescence signal along with the free ligands remaining in the solvent, each contributing at a certain fluorescence quantum yield (QY). The curve associated with the black squares corresponds to samples with only h-FTAA/buffer, used as a reference signal (QY 5%). It can be concluded from the simulations that the primary binding site for huWT has a high quantum efficiency, 43%, compared with 17% for that of the moWT. When the second h-FTAA binds in proximity, the total QY of the system drastically reduces to approx. 2% for both the mouse and human variants. Both the human and mouse binding sites are similar in binding strength (k_d_ = 20 nM and 30 nm, respectively). Figure 4 also includes plots for native (non-fibrillar) αsynuclein in buffer (pink and magenta solid circles), showing very similar results for both the human and mouse variants, with a moderate QY of 22%. However, the native protein binding affinity is substantially weaker, with dissociation constants of approximately 650 nM, more than 20-fold higher than the primary binding sites on the PFFs.

Conclusively, our binding studies show that huWT and moWT αsyn PFFs exhibit similar binding affinities for h-FTAA, with k_d_ in the 20–30 nM range. Despite this similarity in binding strength, h-FTAA demonstrates substantially higher fluorescence QY when bound to the primary sites of huWT PFFs, indicating a more hydrophobic, rigid binding pocket that restricts molecular rotation of the fluorophore. Conversely, the lower QY observed with moWT PFFs suggests a more flexible or hydrophilic binding environment. Thus, the distinct fluorescence properties observed between human and mouse PFFs reflect fundamental differences in their fibrillar architectures. These findings align with previous studies demonstrating species-specific binding preferences for various amyloid-detecting dyes. More specifically, the preference of hydrophilic dyes (FSB) [36] for mouse fibrils and hydrophobic dyes (ThT [15], Nile Red [37], and h-FTAA) for human fibrils indicates fundamental differences in surface hydrophobicity between these structurally related but functionally distinct amyloid assemblies. These binding characteristics have significant implications for confocal microscopy studies due to the differential visualization efficiency between human and mouse αsyn aggregates depending on the fluorophore employed. Furthermore, these findings highlight the importance of considering species-specific differences in translational research for the validation of diagnostic detection probes or therapeutic compounds targeting αsyn aggregates, as ligands optimized for human pathology may perform differently in mouse models of synucleinopathies.

### 2.5. Confocal Microscopy and FLIM of h-FTAA-Labeled hu-or Moαsyn PFFs

To obtain a more nuanced characterization of fibril morphology, imaging techniques involving spectral and fluorescence lifetime imaging microscopy (FLIM) were applied complementarily to assess the local photophysical microenvironment surrounding fibrils in vitro (for details, see Section 3.7). To this end, in vitro moWT-/huWT PFFs were stained with varying concentrations of h-FTAA. For characterizing moWT PFFs, the fibril was fixed at 25 µM, while the h-FTAA concentrations were varied to 2.5, 5 and 10 µM, yielding h-FTAA:protein ratios of 1:10, 1:5 and 2:5. For assessing huWT PFFs, two concentrations of h-FTAA were applied, 2.5 µM and 5 µM, with the fibril concentration maintained at 25 µM. Representative fluorescence images of in vitro h-FTAA-stained huWT-/moWT PFFs are presented in Figure S3. Drawing inference from the previous section, it was observed that the quantum yield for in vitro binding of h-FTAA to moWT PFFs was much lower than that observed for h-FTAA-bound huWT PFFs. h-FTAA, when bound to mouse fibrils, possibly adopts a more twisted, less planar configuration than when bound to human fibrils, producing both a blue-shifted emission and reduced quantum yield, possibly due to shortened pi-electron conjugation and enhanced non-radiative decay. These photophysical differences likely reflect subtle structural variations in sequence-specific fibril packing between mouse αsyn fibrils [15], which may influence how tightly h-FTAA can planarize on the fibril surface

**Figure 5:**
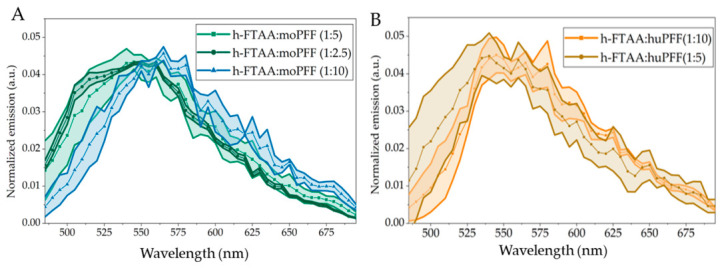
Spectral emission profiles of h-FTAA binding to (**A**) moWT PFFs and (**B**) huWT PFFs fixed at 25 µM with varying concentrations of h-FTAA: 2.5, 5 or 10 µM. Inset: ligand:protein ratios. The shaded region in each plot represents the standard deviation from different ROIs in each experiment. λ_ex_ = 475 nm, and emissions were collected in the range λ_em_ = 490–700 nm.

Spectral emissions were collected by exciting samples at 475 nm, and the corresponding emission profiles were generated by selecting different regions of interest post-background correction. The shaded regions in the spectral emission plots shown in Figure 5 represent the standard deviation from four ROIs obtained from each experiment. Notably, the emission profiles generated at h-FTAA loading concentrations of 5 and 10 µM, upon binding to moWT PFFs, exhibit broad emission maxima at ~505–550 nm, with blue-shifted features (Figure 5A). Meanwhile, at a lower conc. of h-FTAA loading of 2.5 µM, the emission spectrum of h-FTAA-bound moWT PFFs shows an emission maximum at ~550–580 nm, with a red-shifted appearance.

The spectral emission plots for h-FTAA binding to huWT PFFs at a 2.5 µM h-FTAA loading, the emission maximum was found to be ~545–580 nm, while at a 5 µM h-FTAA concentration, the emission maximum was observed to be ~535–565 nm, relatively showing a trivial, blue-shifted tendency (Figure 5B). This observation of h-FTAA exhibiting red-shifted emission upon binding to huWT PFF coincides well with our previous findings [21,23].

**Figure 6:**
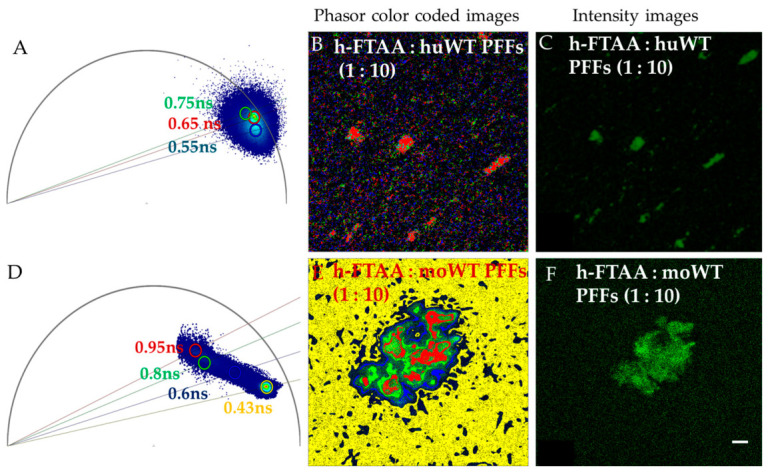
Representative phasor FLIM plots and corresponding phasor color-coded and fluorescence intensity images of 2.5 µM of h-FTAA binding to 25 µM of (**A**–**C**) huWT PFFs and (**D**–**F**) moWT PFFs. λex = 475 nm. Scale bar represents 10 µm.

Next, FLIM images were recorded for the same samples, which were used for spectral emission analysis, as shown in Figure 6. Several ROIs were selected for analyzing fluorescence lifetime profiles in those same stained regions, which were used for spectral analysis. To characterize the fluorescence lifetime profiles of h-FTAA fibrils, we applied two complementary analytical approaches: phasor FLIM analysis and exponential decay fitting. Phasor FLIM analysis is a rapid, fit-free method to analyze the fluorescence lifetime of heterogeneous complexes in real time. This method transforms each pixel’s decay into a point in the phasor plot, allowing direct identification of distinct fluorescence lifetime populations indicative of fluorescence dye binding milieu. The fluorescence lifetime distributions of h-FTAA-labeled huWT PFFs or moWT PFFs at a 2.5 µM ligand loading are shown as phasor plots in Figure 6A,D, with corresponding phasor color-coded images presented in Figure 6B,E.

As observed from the phasor plots, the h-FTAA-labeled human WT PFFs exhibit a narrower fluorescence lifetime distribution (0.65–0.75 ns) compared with mouse WT PFFs (0.6–0.95 ns) at a 2.5 μM ligand concentration. This broader distribution in mouse PFFs indicates a greater structural variability in binding pockets, potentially reflecting a more heterogeneous fibril population, as reflected by the varied fluorescence lifetime populations within the phasor plots.

At a 5 µM h-FTAA loading, the h-FTAA-probed huWT PFFs exhibit slightly longer fluorescence decay distributions ranging from ~0.8 to 1ns (Figure S4A,B), while h-FTAA-labeled moWT PFFs exhibit fluorescence decay distributions of ~0.9–1 ns (Figure S4D,E). At a 10 µM h-FTAA loading, the h-FTAA-labeled moWT PFFs exhibit fluorescence decay distributions of ~1.0–1.5 ns, as shown in Figure S5. This concentration-dependent shift likely reflects the progressive saturation of primary binding sites and increasing occupation of secondary binding sites with different photophysical properties.

A complementary FLIM analysis using exponential decay fitting is presented in Figure S6A,B, supporting the results presented above based on the phasor FLIM approach, showing longer fluorescence decay profiles with higher loading concentrations of h-FTAA. The distinct phasor distributions between αsyn variants show that fundamental differences in fibril microarchitecture influence the photophysical properties of bound h-FTAA, further confirming that the differences observed in QY between human and mouse PFFs stem from distinct molecular environments rather than simply different binding affinities. These differences in fibril architecture could influence not only dye-binding properties but also interactions with cellular components, potentially explaining species-specific differences in seeding efficiency, toxicity, and propagation.

### 2.6. Confocal Microscopy and FLIM of h-FTAA-Probed αsyn Aggregates in Rat Heart Tissue Sections, Induced by Gut-First hu- or moPFF αsyn Onset

Peripheral synucleinopathy and cardiac denervation are key features of PD and DLB, often occurring years before motor symptoms [26]. Prodromal PD and peripheral synucleinopathy are commonly modeled via injection of αsyn PFFs into peripheral organs like the gut. To translate our in vitro imaging methodology into in vivo settings, we examined αsyn aggregates in rat heart tissue sections following a gut-first seeding paradigm [38] with either human or mouse PFFs (see Section 3.8 and Section 3.9 for details). The PFFs were injected into the upper duodenum and pyloric region in 14-month-old wild-type (Fisher 344) rodents, and subsequent pathology in cardiac tissue was assessed at 6.5 months post-injection (mpi).

**Figure 7:**
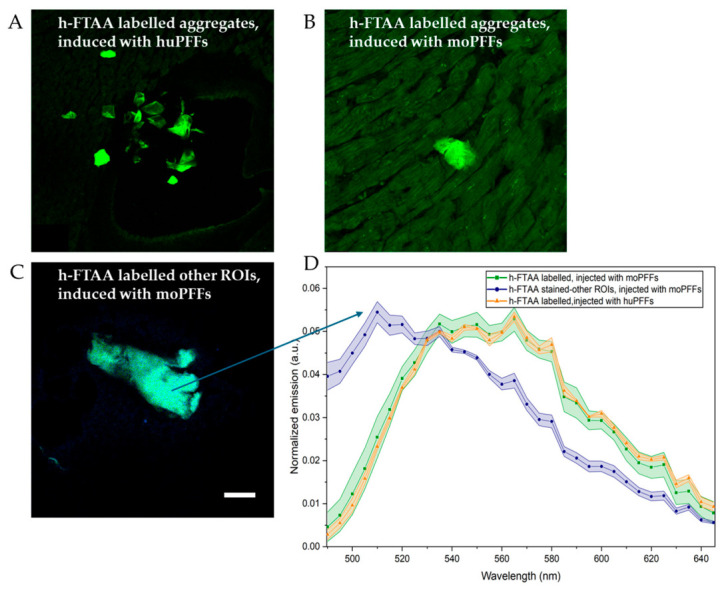
Representative fluorescence intensity images of h-FTAA-labeled αsyn aggregates in rat heart tissue sections and corresponding spectral emission profiles. (**A**) Pathology in huPFF-seeded rodents, (**B**) pathology in moPFFs-seeded rodents, and (**C**) selected ROIs showing distinct patterns different from the ones seen in (**B**). (**D**) Spectral emission profiles of h-FTAA binding to aggregates. λex = 475 nm, and emissions were collected in the range λem = 490–700 nm. The blue arrow indicates the representative ROIs used for generating the emission plot, shown in blue. The shaded region in the plot represents the standard deviation from different ROIs used in each experiment. Scale bar represents 25 µm.

Figure 7 presents representative confocal images of h-FTAA-labeled αsyn aggregates in rat heart sections. Cardiac aggregates from huPFF-injected rodents (Figure 7A) exhibit enhanced fluorescence intensity with distinct cluster formation and minimal background interference. In contrast, cardiac aggregates from moPFF-injected rodents (Figure 7B) display comparatively reduced fluorescence intensity with higher background interference. Additionally, select ROIs reveal that aggregates from moPFF-seeded rodents occasionally presented with diffuse, cloud-like patterns (Figure 7C) that were morphologically distinct from those observed in Figure 6B. Furthermore, the spectral emissions were scanned by exciting samples using a 475 nm laser line. Spectral emission profiles were constructed by selecting different ROIs and correcting the background to reduce non-specific signals. The shaded regions in the emission profiles in Figure 7D represent standard deviations from different ROIs in each section. The emission profiles of h-FTAA upon aggregate-binding yield a characteristic double peak with maxima around ~540–580 nm, showing a red-shifted shoulder toward the end of the spectrum. This observation resonates with the emission maxima usually observed for h-FTAA when bound to pathological αsyn inclusions in brain tissue sections of PD and MSA patients [24]. Strikingly, this observation also coincides with our previous study involving a cell-model-based investigation of αsyn aggregate morphology using h-FTAA [23]. Additionally, we observed distinct emission spectra with emission maxima at ~510–535 nm, a blue-shifted feature (Figure 7D) in a few selected ROIs in rat heart tissue sections of moPFF-seeded rats (Figure 7C). These distinct spectral profiles coincide with the in vitro spectral emission profiles of h-FTAA-labeled moPFFs (see Section 2.3).

**Figure 8:**
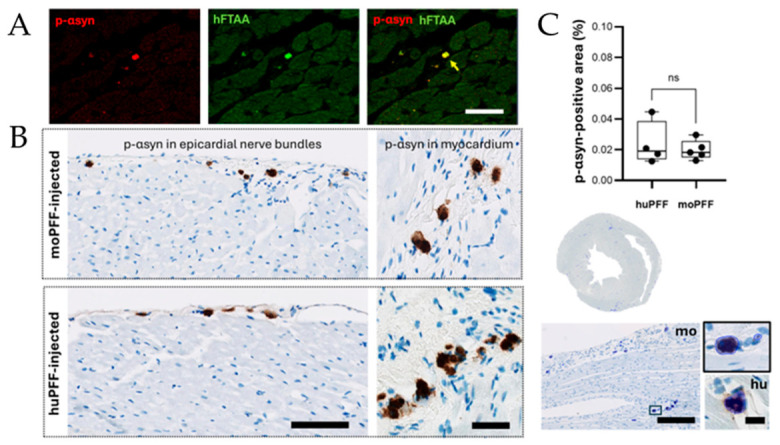
(**A**) Double immunofluorescence staining of h-FTAA with pSer129 αsyn antibody (Ab51253, Abcam) shows co-localization, verifying binding of h-FTAA to deposits positive for phosphorylated αsynuclein (p-αsyn) (indicated in yellow). Scalebar: 200 µm. (**B**) Phosphorylated αsyn pathology in heart of gut-first seeded rats following mouse PFF (moPFF) or human PFF (huPFF) injection. Representative immunohistochemical images showing p-αsyn accumulation (brown) in epicardial nerve bundles (left panels; scalebar: 100 µm) and myocardium (right panels; scalebar: 50 µm) of gut-seeded rats injected with moPFF or human PFF. Nuclei are counterstained in blue. (**C**) Quantification of p-αsyn-positive area normalized to total tissue area in cardiac sections from moPFF- and huPFF-injected rats, using an automated detection algorithm, developed using Aiforia software. Blue regions in the images demonstrate automated detection of p-αsyn-positive deposits (>5 µm^2^). No significant difference (ns) was observed between groups at 6.5 months post-injection. Scalebars: 200 µm and 25 µm for high-magnification images.

Double immunofluorescence staining confirmed precise co-localization of h-FTAA with phosphorylated (pSer129) αsyn (p-αsyn), validating the probe’s specific binding affinity for pathogenic αsyn aggregates (Figure 8A and Figure S8). The blue shift observed in h-FTAA fluorescence of some cardiac deposits of moPFF-seeded rodents likely reflects a structural difference in these aggregates. More densely packed β-sheet arrangements can modify the electronic environment of bound h-FTAA, resulting in a shift toward shorter wavelengths. Given that moPFFs promote faster αsyn accumulation and propagation in rodents, it is plausible that moPFF-injected animals harbor more mature and densely packed deposits compared with huPFF-injected rodents, causing the observed spectral shifts in some deposits. Immunohistochemical (IHC) detection of αsyn aggregates cannot distinguish between different conformations or packing density, confirmed by our IHC-based detection of αsyn pathology (Figure 8A–C). IHC was performed on neighboring sections used for h-FTAA-based characterization, using an antibody against p-αsyn and proteinase pretreatment to ensure removal of endogenous protein. We did not observe any difference between the p-αsyn-positive area in the heart of moPFF- vs. huPFF-injected rodents at 6.5 mpi. This is not surprising as quantification of αsyn burden alone correlates poorly with disease severity due to ongoing neurodegeneration [39]. Therefore, IHC-based scoring of peripheral biopsies is unlikely to reliably distinguish between disease subtypes and stages. In contrast, h-FTAA has the advantage of binding a broad spectrum of protein conformers, not limited to phosphorylated species [40,41,42,43,44] like pSer129. Figure S8A,B illustrates h-FTAA’s ability to detect more extensive pathology compared with the p-αsyn antibody binding.

In this study, the in vivo hFTAA-based characterization of peripheral αsyn aggregation is limited to the late disease endpoint, as the timing of pathology emergence in the peripheral nervous system remains uncertain. However, in the context of biomarker development, it is essential to consider the temporal evolution of αsyn aggregation, as the stage of aggregation may directly influence disease-specific diagnosis and prognosis.

The amyloidogenic targets for h-FTAA binding have been very clearly mapped for amyloid-β in vitro and in APP transgenic mice and human samples in vivo, where h-FTAA binds to most types of aggregates with fibril conformational properties (early on-pathway oligomeric and early and mature fibrils) [41,42,43,44]. Interestingly, one study suggested that the red-shifted spectral peak observed with h-FTAA may correspond to earlier or more immature amyloid-β deposition [41]. The h-FTAA targeting has not been as thoroughly mapped for αsyn aggregates, but it is clear that h-FTAA binds early aggregates (prior to ThT) and also to late fibrils in vitro [21]. Aged wild-type Fisher 344 rats naturally develop αsyn aggregates in the peripheral nervous system as they age. Interestingly, we observe a more heterogeneous spectral range of h-FTAA-bound deposits in the healthy aged heart. The more lightly stained deposits (usually antibody-negative) show a more red-shifted h-FTAA spectral read-out (Figure S8C,D). It is conceivable that the conformational mix detected in the aged wild-type rodent heart represents a mixture of mature and immature αsyn deposits rather than a mix of distinct αsyn strains. This observation underscores the potential of h-FTAA to inform on aggregate maturity in synucleinopathies, similar to reports from amyloid-β studies. The next essential step will be to investigate early peripheral pathology in relevant synucleinopathy models to better understand the significance of h-FTAA spectral shifts at the prodromal disease stage.

**Figure 9:**
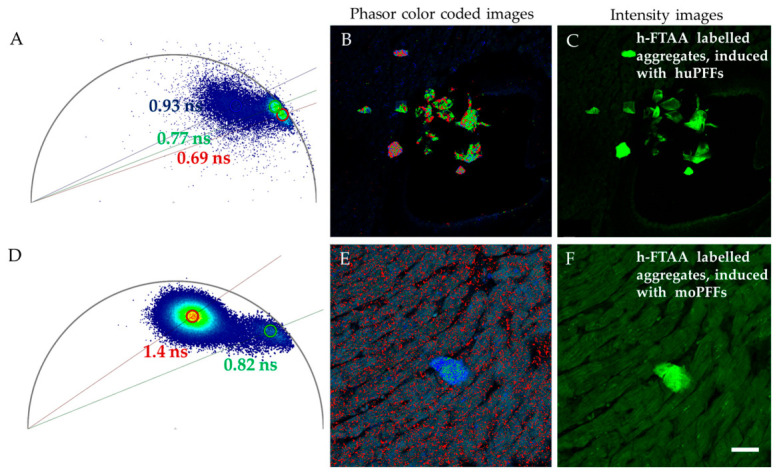
Representative phasor FLIM plots and corresponding phasor color-coded and fluorescence intensity images of h-FTAA-labeled aggregates in rat heart tissue sections, with pathology induced by (**A**–**C**) huPFFs and (**D**–**F**) moPFFs. λex = 475 nm. Scale bar represents 25 µm.

**Figure 10:**
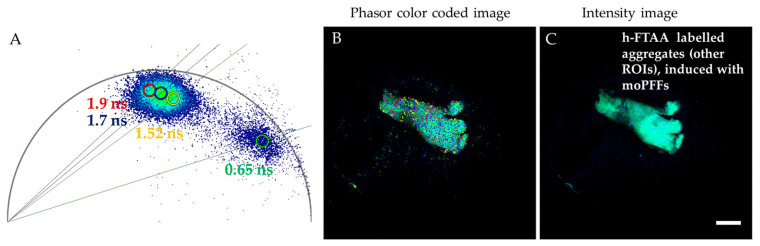
Representative phasor FLIM plots and corresponding phasor color-coded and fluorescence intensity images of distinct ROIs, and h-FTAA-labeled aggregates with pathology induced by (**A**–**C**) moPFFs. λ_ex_ = 475 nm. Scale bar represents 25 µm.

Next, FLIM images were recorded for the same samples that were used for spectral analysis in Figure 7. The same ROIs were selected for assessing the fluorescence lifetime distributions of h-FTAA binding to aggregates, induced by mo- or huPFFs. Phasor FLIM and exponential decay fit approaches were used for analyzing fluorescence lifetime data. Representative phasor color-coded FLIM images and the corresponding phasor plots are depicted in Figure 9A,B,D,E and Figure 10A,B. The fluorescence decay distribution of h-FTAA binding to aggregates in huPFF-injected rodents corresponds to ~0.69–0.8 ns (Figure 9A,B). This finding correlates with our previous study involving cell models to investigate αsyn aggregate morphology, wherein the fluorescence lifetime distribution of h-FTAA binding to intracellular αsyn aggregates, induced by huPFFs, corresponded to ~0.7–0.86 ns [23]. Furthermore, the fluorescence decay distribution of h-FTAA binding to aggregates in moPFF-injected rodents was found to be ~1–1.4 ns (Figure 9D,E), much longer than that of h-FTAA-bound aggregates from huPFF-injected rodents. Exponential decay fit for analyzing FLIM was applied as a complementary approach (Figure S7), which supports the above-presented results for FLIM.

Few ROIs in moPFF-injected rodents harbored h-FTAA-bound aggregates with an additional distinct h-FTAA fluorescence lifetime signature at ~1.5–1.9 ns, as shown in Figure 10A,B. The fluorescence decay distributions of h-FTAA binding to these distinct deposits are longer than those characterized in Figure 9D,E. These differential fluorescent signatures are particularly relevant for evaluating the potential of h-FTAA ligand as a strain-specific biomarker for disease-specific diagnosis and strain-specific intervention across synucleinopathies.

## 3. Materials and Methods

### 3.1. αsyn Protein/PFFs and LCO Materials

Full-length wildtype human or mouse αsyn was expressed in BL21(DE3)-competent cells and purified by ion-exchange and reversed-phase chromatography, as previously described [14,45]. Immediately before PFF generation, purified recombinant monomeric αsyn was passed through a 100 kDa filter to remove unwarranted oligomeric species. The monomeric αsyn (1 mg/mL) was then seeded with sonicated wt mouse or wt human PFFs (produced previously [46,47]) at a concentration of 5% PFFs by mass. The sample was incubated at 37 °C in phosphate-buffered saline (PBS, Gibco, Thermo Fisher Scientific, Roskilde, Denmark) (pH 7.4) with continuous shaking at 1050 rpm in a tabletop microtube shaker (Eppendorf, Thermotop, Sigma-Aldrich, Søborg, Denmark) for 72 h. The fractions were tested for ThT fluorometry and sedimentation analysis to validate amyloid structure and insolubility, as previously described [48]. The validated PFFs were harvested by centrifugation at 15,600× *g* for 30 min into pellet insoluble fibrils and then resuspended in PBS to a concentration of 1 mg/mL, determined using a Pierce BCA protein assay (Thermo Fisher Scientific, Roskilde, Denmark). The PFFs were then sonicated for 20 min with 30 ms pulses, followed by 70 ms breaks at 30% power using a Branson SFX250 Sonifier equipped with a 1″ cup horn (Branson; 101-147-046, Merck Life Science, Søborg, Denmark) and stored at −80 °C.

h-FTAA was received from Prof Peter Nilsson’s group at the Department of Physics, Chemistry and Biology, Linkoping University, Linkoping, Sweden.

### 3.2. Aggregation Kinetics and Cross-Seeding Experiments with huWT and moWT

The protein concentration was quantified with a photometer (NanoPhotometer NP80, IMPLEN (Munich, Germany)), using the extinction coefficient, 5960 M^−1^·cm^−1^ for human and 7450 M^−1^·cm^−1^ for mouse a-syn [29], at 280 nm. For kinetics analysis, native huWT and moA53T a-syn were incubated with either ThT in PBS to a final protein concentration of 30 μM. The fluorophore concentration was 10 μM for ThT prepared in a final volume of 50 μL and pipetted in quadruplicate into a 96-well plate (Corning, Oslo, Norway,96-Well Half Area Black/Clear Flat Bottom Polystyrene NBS Microplate, 3881). The outer two rows and columns were deliberately not used, as samples in these wells are prone to evaporation. PBS-only wells and native protein in PBS were used as controls. The plate was transported into the BSL-3 lab, where another control of already formed a-syn fibrils was added for calibration. One 3 mm glass bead (Sigma-Aldrich, Merck Life Science AS, Oslo, Norway, Solid-glass beads, Z265926) was added to each well containing a sample to promote agitation and fibrillation. MilliQ (MQ) water was added to adjacent wells, as well as in between wells, to protect samples from evaporation. The well plate was sealed with aluminum foil before it was incubated in the Clariostar plate reader (BMG Labtech, CLARIOstar Plus, Oretenberg, Germany,) at a temperature of 37 °C and under agitation (5 min at 600 rounds per minute (rpm)). The aggregation process was monitored in real time by collecting fluorescence every 15 min for 40 h with the settings: excitation at 440 nm; emission at 470–640 nm. Emissions from “probe-only” samples were used as a baseline. The spectral properties of the samples and control PPFs were further analyzed using a Tecan Saphire^2^ fluorescence plate reader (Tecan, Männedorf, Switzerland).

### 3.3. Data Analysis of the Fibril Aggregation and Cross-Seeding Assays

The raw data in terms of fluorescence spectra from each well were integrated, and the resulting intensity vs. time signal was smoothed using a standard 13-point, 3rd-order, Savitzky–Golay moving average filter. The time profiles were baseline-corrected with the PBS/dye-only sample data prepared in an identical manner, and after removing evident outliers, the average signal of each sample set was calculated and re-normalized for convenient data fitting and comparison in the associated plots. The time profile of the aggregation kinetics was fitted using the widely used sigmoidal function [48,49]:

(1) $$F\left(t\right)=F_{0}+\frac{F_{max}-F_{0}}{1+exp\left[-k_{app}\left(t-t_{1/2}\right)\right]}\;$$

where $F\left(t\right)$ is the collected time series of fluorescence data, and $F_{0}$ and $F_{max}$ are the initial and final plateau of the sigmoidal curve shape, respectively. The essential parameters for the aggregation kinetics are contained in the parameters $k_{app},$ the apparent growth rate (units of time^−1^), and the time point $t_{1/2},$ defining the inflection point of the sigmoidal shape (50% of $F_{max}-F_{0}$), usually referred to as the half-time value. These can then be used to calculate the so-called lag-time, $t_{lag}=t_{1/2}-\frac{2}{k_{app}}$, defining the time point when significant aggregation begins.

Similarly, the time profile of the seeding aggregation kinetics showed, in two cases (Figure 2), a biphase behavior and was fitted to a linear combination of the function, as shown in Equation (1):

(2) Ft=frac1+exp−kapp 1t−t1/2 1+1−frac1+exp−kapp 2t−t1/2 2 

To reduce the number of free parameters in the fitting, the data points were re-normalized to have $F_{0}=0$. The essential kinetic parameters, along with standard deviation via the quality of the fit, are presented in Table 1 in the Section 2.1 and Section 2.2, along with further details for the different cases. The data manipulation, fitting and presentation were carried out using Origin Pro 2025.

### 3.4. Transmission Electron Microscopy Analysis

Mo- and huPFFs were thawed and imaged using TEM. Here, the PFF stock solution (70 µM on a monomer basis) sample was applied to the TEM grid to adsorb for 2 min (400-mesh copper grids CARBON-B, Ted Pella Inc., Redding, CA, USA). The excess sample was blotted off with a filter paper, and the grid was washed with one round of milli-Q water and was negative-stained with 2% uranyl acetate (water solution) for 30 s. The grids were blotted dry and dried in air. Imaging was performed using a Jeol JEM1400 Flash TEM instrument (JEOL Nordic AB, Hammerbacken, Sweden) operating at 80 keV, with images taken at 5000× and 25,000× magnifications.

### 3.5. Dynamic Light Scattering

The hydrodynamic size distribution profile of the sonicated PFF was measured by dynamic light scattering (DLS) with a Wyatt DynaPro NanoStar instrument (Waters Corporation, Santa Barbara, CA, USA) at 25 °C. Each sample was analyzed in triplicate with ten measurements of 5 s, each averaged for each histogram. The data were processed using the Dynamics 7.5.0.17 software package with the solvent (PBS) background signal subtracted from each sample.

### 3.6. Emission of αsyn PFFs Together with h-FTAA and Simulation of Binding Curves

For spectral assessment and binding curves, the αsyn PFFs were diluted to 1 µM (on a monomer basis) in PBS and mixed with varying concentrations of h-FTAA, ranging between 0 and 4500 nM. Following this, they were incubated overnight at room temperature in a black 96-well plate with a clear bottom (Corning 8085, Fisher Scientific). The excitation and fluorescence spectra were measured using a Tecan Saphire 2 plate reader using excitation spectra of 400–520 nm (emission: 550 nm) and emission spectra of 500–700 nm (450 nm excitation) with 5 nm steps and slits. The one- and two-site-binding models, used to simulate binding kinetics, were developed to calculate the relative abundance of free ligands, empty protein sites, and the protein filled in the one and two sites (see [32] for further details). It should be emphasized here that the binding curves do not follow an analytical expression for the various samples (fibrils and native αsyn), since each simulated curve is a superposition of the fluorescence of the bound ligand together with the free ligand, as deduced from the kinetic model, each with its unique and different quantum efficiency. Therefore, a fitting procedure could not be performed. The SD values of the experimental raw data (triplicates) are indicated with error bars in the plots.

### 3.7. h-FTAA Labeling of In Vitro mo- and huPFFs for Hyperspectral Analysis and FLIM

The hu-/moPFFs fixed at 25µM were mixed with varying concentrations of h-FTAA (2.5, 5 or 10 µM), followed by overnight incubation. The contents were then spun at 13,000 rpm using centrifugation. Subsequently, the harvested pellets (~2 µL) were added onto the microscopy slides. The samples were dried and then covered with coverslips. The pre-stained PFFs were assessed for spectral features and FLIM using a Leica Stellaris 8 FALCON confocal microscope with LAS X software (Leica Microsystems, GmbH, Wetzlar, Germany). The integrated LAS X software, was used for phasor, exponential decay fitting-based FLIM measurements and data analysis. The samples were excited at 475 nm, and the emissions were collected from 490 to 700 nm, divided into 43 steps with a 5 nm step size.

### 3.8. Immunohistochemical Detection of Phosphorylated Alpha-Synuclein

Paraffin-embedded heart sections of gut-first PD rodents were provided and stained by N.V.D.B. and V.T. Automated IHC was performed using p-αsyn antibody (D1R1R, Cell Signaling Technology, distributed by Bionordika, Herlev, Denmark, 1:1000–with proteinase pretreatment) on the Discovery autostainer (LaRoche Diagnostics, Copenhagen, Denmark) [50,51]. Processed slides were scanned using automated microscopy (S60, Visiopharm, Hørsholm, Denmark). Automated detection and quantification of the p-αsyn-positive area normalized to the total tissue area was performed using the Aiforia software (https://www.aiforia.com/, v7.0).

### 3.9. h-FTAA-Based Detection of Phosphorylated Alpha-Synuclein

The binding specificity of h-FTAA to p-αsyn-positive aggregates was verified via manual double immunofluorescent staining. The tissue sections were deparaffinized and rehydrated following standard protocols. The sections were blocked with 5% normal goat serum (blocking buffer) for 30 min at room temperature to minimize non-specific binding. After blocking, the sections were incubated with anti-pSer129 αsyn antibody (Ab51253, Abcam, Cambridge, UK, 1:1000 in blocking buffer) overnight at room temperature. Following three 5 min washes with PBS-T, the sections were incubated with Alexa647-conjugated secondary antibody (Thermo Fisher Scientific, Roskilde, Denmark, 1:400 in blocking buffer) for 1 h at room temperature. After thorough washing, the sections were incubated with h-FTAA (0.5 μM) for 30 min, followed by DAPI counterstaining for nuclear visualization. Colocalization of h-FTAA (green fluorescence) with pSer129-positive inclusions (red fluorescence) was visualized using confocal microscopy (inverted Zeiss LSM800 Airyscan with ‘Zen Blue’ software (Carl Zeiss AG, GmbH, confirming the probe’s selective binding to pathogenic αsyn deposits in cardiac tissue.

For spectral and lifetime characterization studies, h-FTAA fluorescence measurements were performed exclusively on single-stained tissue sections to prevent potential spectral interference from antibody-conjugated fluorophores, ensuring accurate acquisition of the probe’s intrinsic photophysical properties when bound to αsyn aggregates.

## 4. Conclusions

This study demonstrates, for the first time, that the h-FTAA ligand can detect peripheral αsyn aggregates in the hearts of PD rodent models, confirmed by IHC. We show that combining h-FTAA spectral emission profiling with fluorescence lifetime imaging reveals distinct conformational states of the ligand when bound to aggregates, reflecting protein architecture and the molecular environment surrounding the protein deposits. Our results indicate that the sequence (species origin) of the seeding material (mouse or human) used to induce gut-first PD in rodents influences the in vivo aggregate conformations, as evidenced by differences in h-FTAA binding characteristics and fluorescence patterns, which may correspond to clinically relevant strain variations in synucleinopathies. Recent advances have shown that αsyn detection in skin biopsies can diagnose synucleinopathies at early disease stages using IHC and seed amplification assays (SAAs). However, these methods cannot distinguish disease-specific strains or identify disease stage. In contrast, the spectral variations observed in peripheral cardiac pathology from moPFF- vs. huPFF-injected rodents in this study indicate the ability of h-FTAA to identify differences in both aggregate conformation and maturity.

Our findings underscore the potential of LCO ligands, like h-FTAA, to improve diagnostic precision by enabling in-depth characterization of peripheral deposits, complementing existing IHC- and SAA-based approaches. Future studies should evaluate h-FTAA’s ability to discriminate αsyn strains in more accessible tissues (e.g., the skin, gut, and liver). Detecting and distinguishing α-syn conformations in peripheral biosamples could reveal disease propagation pathways and enable earlier, disease-specific classification prior to the onset of motor symptoms [52].

Because aggregate burden alone correlates poorly with clinical severity and phenotype in synucleinopathies, there is a critical need for biomarkers that inform additional pathological features [53,54,55]. LCO ligands, like h-FTAA, may fulfil this role by concurrently assessing aggregate conformation and maturity in a single assay, facilitating more comprehensive assessment of disease stage, prognosis, and therapeutic targeting.

## Figures and Tables

**Table 1:** Parameters of the aggregation kinetics using ThT deduced from fitting of the time lapses in Figure 1 ^1^.

Sample	$k_{app}$ (h^−1^)	$t_{1/2}$ (h)	$t_{lag}$ (h)
huWT 30 μM	0.166 ± 0.06	37.5 ± 0.2	25.5 ± 0.9
moWT 30 μM	0.978 ± 0.002	11.1 ± 0.03	9.05 ± 0.03
huWT 50 μM	0.310 ± 0.008	29.1 ± 0.1	22.6 ± 0.6
huA53T 48 μM A	0.898 ± 0.004	19.8 ± 0.1	17.6 ± 0.1

^1^ For more details on 50 µM of huWT and 48 µM of huA53T, see reference [21].

**Table 2:** Parameters of the seeding aggregation kinetics using ThT deduced from fitting of the time lapses in Figure 2.

Sample	kapp1 (h^−1^)	kapp2 (h^−1^)	t1/21(h)	t1/22(h)
huWT + huPFF	1.8 ± 0.3	1.8 ± 0.5	4	1
moWT + moPFF	3.6 ± 0.6	-	0.44 ± 0.05	-
huWT + moPFF	0.32 ± 0.03	0.72 ± 0.06	19.4	5.7
moWT + huPFF	3.34 ± 0.08	-	0.90 ± 0.05	

The superscript indices 1 and 2 in the heading represent the two components of the biphase model as described by the corresponding parameters in Equation (2) (Section 3.3).

## Data Availability

The original contributions presented in this study are included in the article/Supplementary Material. Further inquiries can be directed to the corresponding author.

## Supplementary Materials

The following supporting information can be downloaded at https://www.mdpi.com/article/10.3390/ijms27093807/s1.
