# Supplementary material for: Human and Mouse Alpha-Synuclein Fibrillation: Impact on h-FTAA Binding and Advancing Strain-Specific Biomarkers in PD Animal Models
Source: Int J Mol Sci. 2026 Apr 24;27(9):3807. doi: 10.3390/ijms27093807 (PMC13163712; doi:10.3390/ijms27093807)
Supplement: Supplementary file 1 [file ijms-27-03807-s001.zip › ijms-4219128-supplementary.pdf]

Supplementary information : Human and Mouse Alpha-Synuclein Fibrillation – Impact on h-FTAA-Binding and Advancing Strain-Specific Biomarkers in PD Animal Models

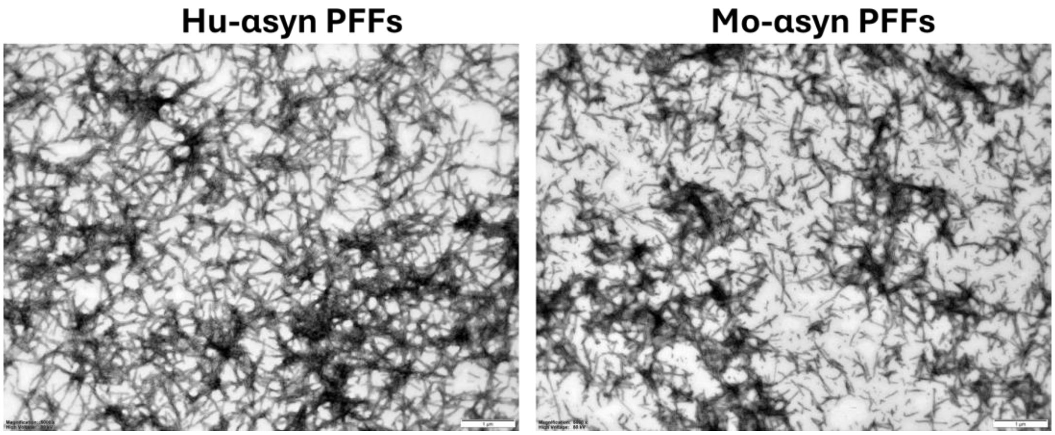

Figure S1: TEM images of huWT- and moWT PFFs at 5.000X magnification, scale bar is 1μm.

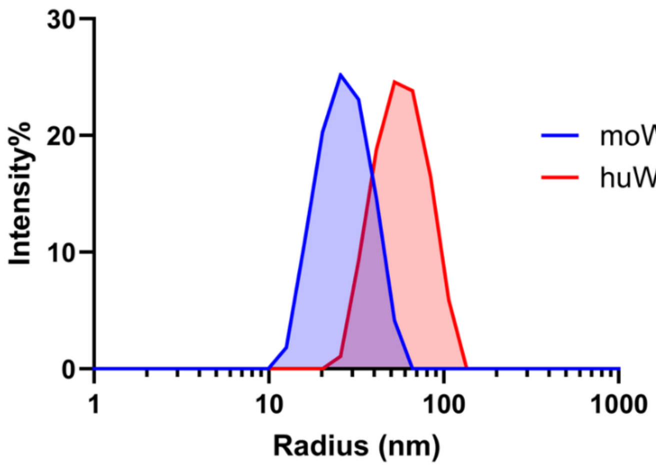

Figure S2: Dynamic light scattering (DLS) analysis of moWT and huWT PFF after fragmentation by sonication. Results are displayed as density curves of the intensity distribution. huWT PFF fragments had a average hydrodynamic radius of 55.2 nm, and moWT PFF had a average hydrodynamic size of 25.7 nm.

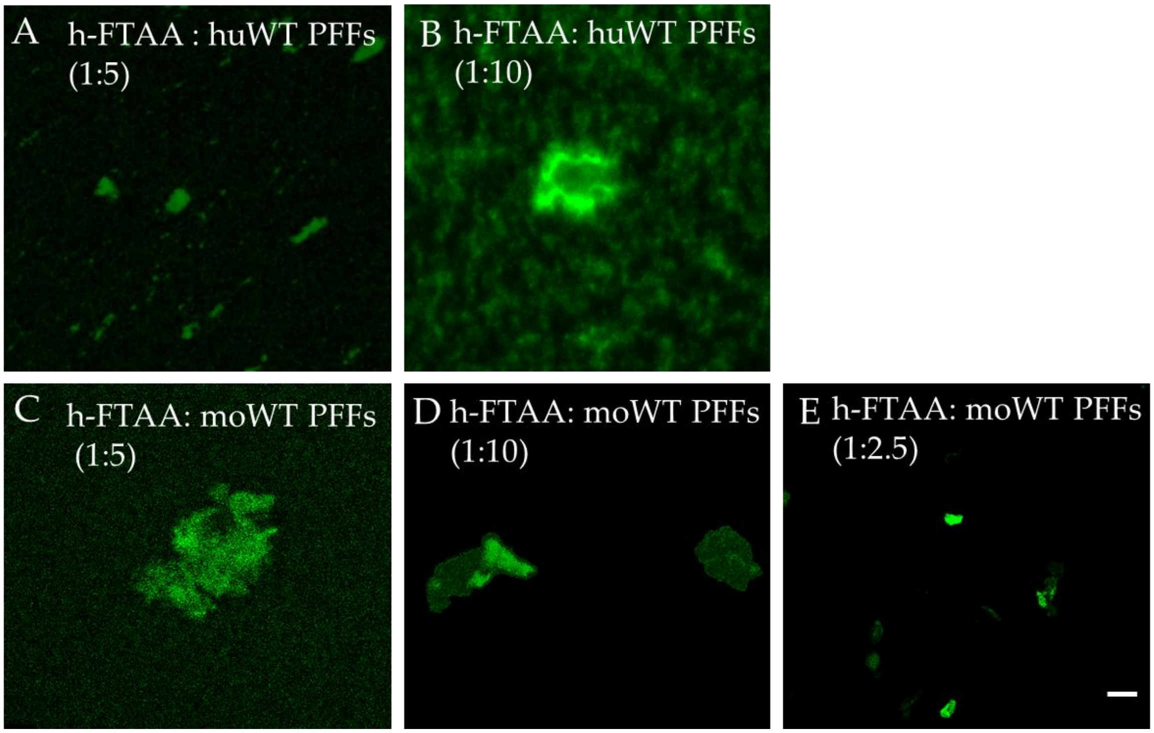

Figure S3: Representative fluorescence intensity images of 25µM huWT PFFs stained with (A) 5µM, (B) 2.5µM of h-FTAA and 25µM moWT PFFs stained with (C) 5µM, (D) 2.5µM, (E) 10µM of h-FTAA. λ_{ex} = 475 nm. Scale bar represents 10µm

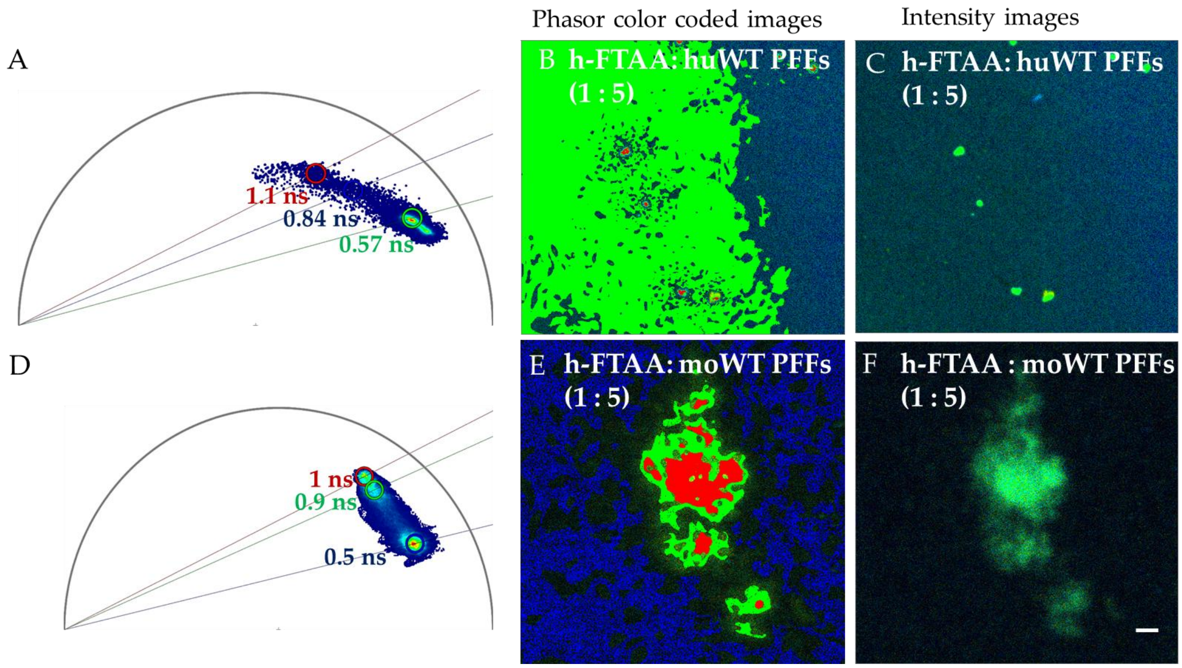

Figure S4: Representative phasor FLIM plots and corresponding phasor color coded and fluorescence intensity images of 5μM h-FTAA- binding to 25μM (A, B, C) huWT PFFs and (D, E, F) moWT PFFs. λ_{ex} = 475 nm. Scale bar represents 10μm.

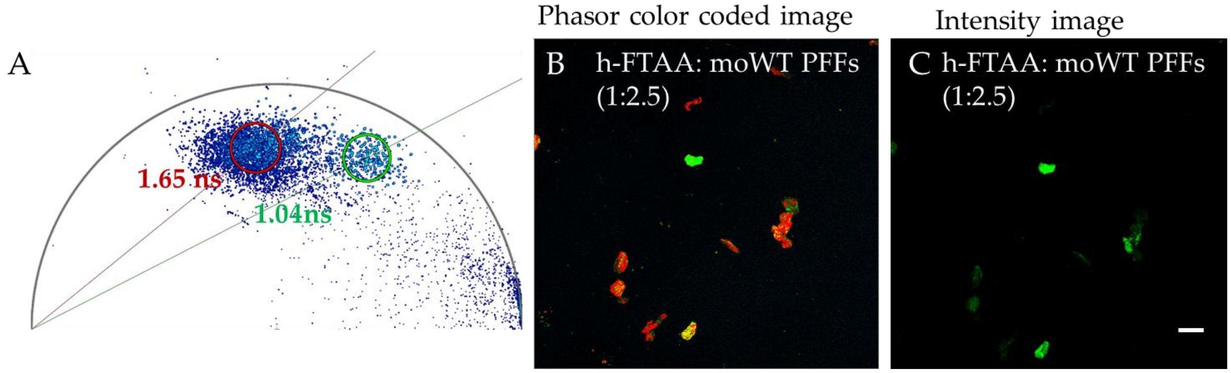

Figure S5: Representative phasor FLIM plots and corresponding phasor color coded and fluorescence intensity images of 10μM h-FTAA- binding to 25μM of (A, B, C) moWT PFFs. λ_{ex} = 475 nm. Scale bar represents 10 μm.

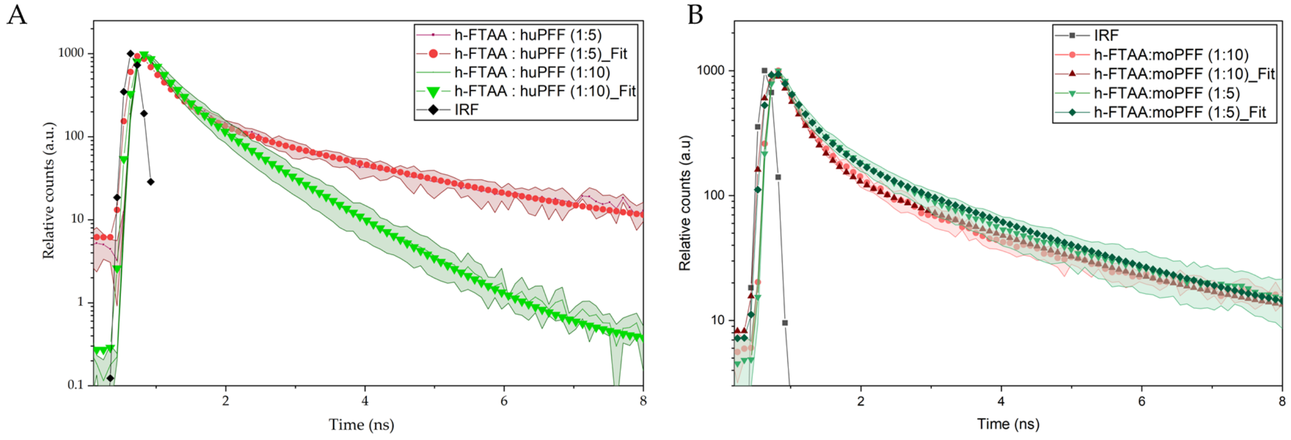

Figure S6: FLIM - exponential decay plots of h-FTAA- labelled (A) huWT PFFs and (B) moWT PFFs. λ_{ex} = 475 nm

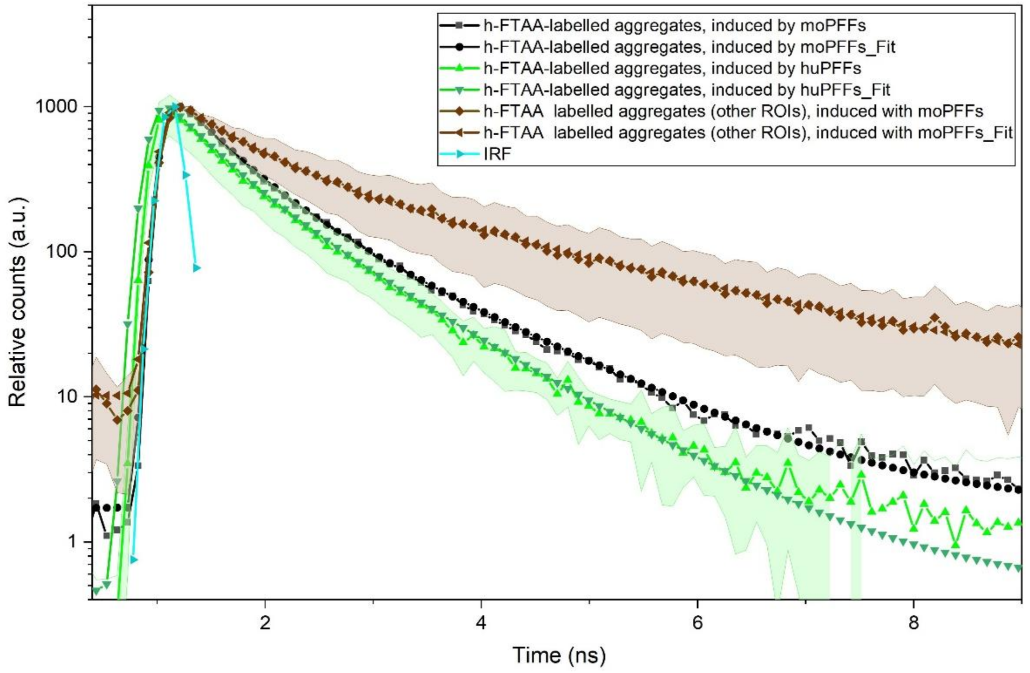

Figure S7: FLIM- exponential decay plots of h-FTAA- labelled- aggregates in rat heart tissue sections. λ_{ex} = 475 nm

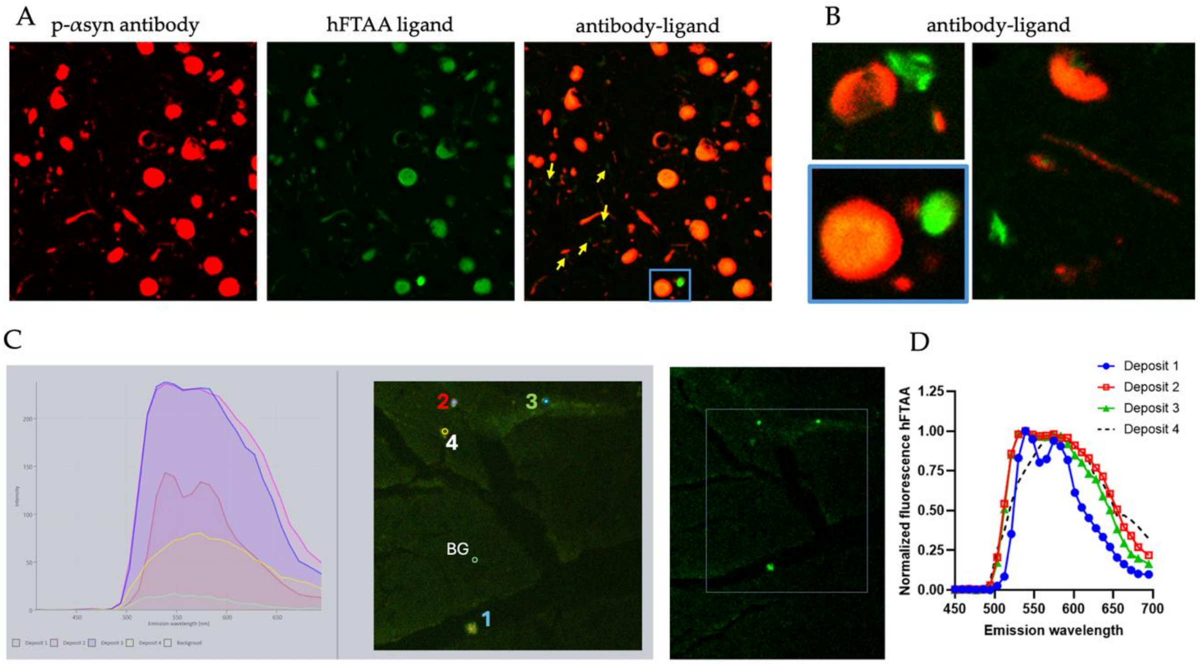

Figure S8 (A) Double immunofluorescence labeling of h-FTAA ligand (green) with pSer129 αsyn antibody (Ab51253, Abcam; red) shows co-localization between h-FTAA and phosphorylated αsynuclein (p-αsyn). h-FTAA co-localizes with nearly all pSer129-αsyn-positive aggregates, indicating high-affinity binding to phosphorylated αsynuclein. Yellow arrows highlight lightly stained h-FTAA-positive, antibody-negative deposits. (B) High magnification images show examples of h-FTAA-positive, antibody-negative deposits, illustrating h-FTAA's ability to detect additional pathology beyond pSer129 immunoreactivity. (C) Example of raw h-FTAA spectral readout of 4 deposits and 1 background (BG) region from a 22-month-old wild-type Fisher 344 rat heart, displaying a heterogeneous mixture of αsyn deposits. (D) Normalized h-FTAA spectra (after background subtraction) of the same 4 deposits. The most lightly stained deposit 4 exhibits a red-shifted spectrum consistent with a putatively less mature conformer.
